# Alcohol and Tobacco Use in an Ethnically Diverse Sample of Breast Cancer Patients, Including Sea Island African Americans: Implications for Survivorship

**DOI:** 10.3389/fonc.2018.00392

**Published:** 2018-09-27

**Authors:** Vivian J. Bea, Joan E. Cunningham, Anthony J. Alberg, Dana Burshell, Colleen E. Bauza, Kendrea D. Knight, Tonya R. Hazelton, Heidi Varner, Rita Kramer, Susan Bolick, Deborah Hurley, Catishia Mosley, Marvella E. Ford

**Affiliations:** ^1^Department of Breast Surgical Oncology, MD Anderson Cancer Center at Cooper, Camden, NJ, United States; ^2^Hollings Cancer Center, Medical University of South Carolina, Charleston, SC, United States; ^3^Department of Public Health Sciences, Medical University of South Carolina, Charleston, SC, United States; ^4^National Coalition of Independent Scholars, San Antonio, TX, United States; ^5^Department of Epidemiology and Biostatistics, University of South Carolina, Columbia, SC, United States; ^6^College of Nursing, Medical University of South Carolina, Charleston, SC, United States; ^7^Department of Hematology/Oncology, Medical University of South Carolina, Charleston, SC, United States; ^8^South Carolina Department of Health and Environmental Control, Columbia, SC, United States

**Keywords:** breast cancer, African Americans, Sea Islanders, alcohol consumption, cigarette smoking, survivorship, recurrence, modifiable risk factors

## Abstract

**Background/Objective:** Data suggest that modifiable risk factors such as alcohol and tobacco use may increase the risk of breast cancer (BC) recurrence and reduce survival. Female BC mortality in South Carolina is 40% higher among African Americans (AAs) than European Americans (EAs). Given this substantial racial disparity, using a cross-sectional survey design we examined alcohol and tobacco use in an ethnically diverse statewide study of women with recently diagnosed invasive breast cancer. This included a unique South Carolina AA subpopulation, the Sea Islanders (SI), culturally isolated and with the lowest European American genetic admixture of any AA group.

**Methods:** Participants (42 EAs, 66 non-SI AAs, 29 SIs), diagnosed between August 2011 and December 2012, were identified through the South Carolina Central Cancer Registry and interviewed by telephone within 21 months of diagnosis. Self-reported educational status, alcohol consumption and tobacco use were obtained using elements of the Behavior and Risk Factor Surveillance System questionnaire.

**Results:** *Alcohol:* EAs were approximately twice as likely to consume alcohol (40%) and to be moderate drinkers (29%) than either AA group (consumers: 24% of non-SI AAs, 21% of SIs; moderate drinkers 15 and 10% respectively). Users tended to be younger, significantly among EAs and non-SI AAs, but not SIs, and to have attained more education. Heavy drinking was rare (≤1%) and binge drinking uncommon (≤10%) with no differences by race/ethnicity. Among both AA subgroups but not EAs, alcohol users were six to nine times more likely to have late stage disease (Regional or Distant), statistically significant but with wide confidence intervals. *Tobacco:* Current cigarette smoking (daily or occasional) was reported by 14% of EAs, 14% of non-SI AAs and 7% of SIs. Smoking was inversely associated with educational attainment. Use of both alcohol and cigarettes was reported by 3–6% of cases.

**Conclusions:** Prevalences of alcohol and cigarette use were similar to those in the general population, with alcohol consumption more common among EAs. Up to half of cases used alcohol and/or tobacco. Given the risks from alcohol for disease recurrence, and implications of smoking for various health outcomes, these utilization rates are of concern.

## Introduction

With the advent of better detection methods and more effective adjuvant therapies, cancer survivorship has steadily increased, with an estimated 3.3 million female breast cancer survivors in the United States as of 2014 (1). However, despite overall increases in breast cancer survival rates, for decades now survival has been lower in African American (AA) women than in European American (EA) women. According to SEER data for 2006–2012, 5-year relative survival from invasive breast cancer was 12% lower in AA's. This disparity in survival is present across all stages of breast cancer, with the least disparity seen with localized disease (5%) but increasing to 13% for regional and 33% for distant stage breast cancer (1). This national disproportionality is also observed in South Carolina, where female breast cancer mortality rates averaged 40% higher among AAs than EAs (28.6 and 20.4/100,000 respectively for 2007–2016) (2). In light of increases in survivorship, research has focused on factors that may affect prognosis and survival and whether these factors contribute to the persistent disparities in mortality.

Alcohol and tobacco use are potential modifiable risk factors for poor breast cancer outcomes. Elevated alcohol intake has been linked to modestly increased risk of recurrence (3), specifically among postmenopausal women (4). However, this is still a controversial association as other studies have either shown no association between alcohol consumption and recurrence and/or mortality (5, 6) or have been inconclusive (7). This discrepancy in findings may be attributable to various aspects of study design and analysis, plus limited mortality events (8). In addition, studies that seek to find a relationship between alcohol consumption and breast cancer have primarily been conducted on European women only (9–11) and can vary depending on the type of alcohol and quantity consumed (12).

Alcohol is now also considered an established risk factor for breast cancer, associated both with a higher likelihood of breast cancer diagnosis and with worse treatment outcomes (8, 13, 14). This relationship to breast cancer etiology and recurrence is not surprising, as alcohol is a known carcinogen (15) through its metabolism to acetaldehyde (16, 17). Acetaldehyde interferes with DNA synthesis and repair, and *in vitro* studies have shown that acetaldehyde causes cytogenetic abnormalities in eukaryotic cells (18, 19). Alcohol-related carcinogenesis and promotion of cancer may also interact with other factors such as smoking, diet, endogenous and exogenous hormones, comorbidities, and genetic susceptibilities and *in vitro* study suggests it may attenuate the effects of Tamoxifen (15, 20–23).

Cigarette smoking may also increase breast cancer mortality among patients who smoke following breast cancer diagnosis and treatment (24). In North Carolina, Parada et al. (25) reported a 54% elevated hazard rate of 13 year smoking-related conditional breast cancer-specific mortality in the Carolina Breast Cancer Study, particularly among AAs. This mortality may be due to increased metastatic potential of breast cancer cells leading to later disease stage diagnoses (26–29). Excess all-cause mortality among breast cancer survivors who smoke has also been reported (24, 30), as smoking is associated with factors leading to poorer health outcomes such as lower socioeconomic status, decreased physical activity and comorbidities (31–34). Genetic factors may also influence susceptibility to carcinogens in cigarette smoke (35). Cigarette smoking may also increase risk for breast cancer among women who consume alcohol (15), particularly if they started smoking before or soon after menarche or have a family history of breast cancer (36). In a recent study of women in North Carolina, risk for breast cancer was found to be increased with long duration of cigarette smoking in AAs only, although potential confounding with alcohol was not examined (37).

In the United States, AAs comprise one of the largest heterogeneous ethnic groups. One sub-population of AAs is the Sea Islanders/Gullah (SIs), descending from West Africans who, skilled in rice farming, were enslaved to work the South Carolina rice plantations in the 1700–1800's (38). The SIs, comprising approximately 250,000 individuals, reside primarily in the fishing and farming communities along the coastal Sea Islands extending along the Atlantic coast from the southern corner of North Carolina through South Carolina into northern Georgia. Their geographic isolation and strong community life have enabled them to preserve more of their African ethnic heritage and genetics than any other historical AA group (38, 39), combining “to produce one of the most distinctive reservoirs of African-American culture in the United States” (40). This is reflected in the distinctive SI dialect, Gullah, with cadences reminiscent of West African languages, still in common use today. Thus, the SI community provides a unique opportunity to further investigate racial disparities in breast cancer in South Carolina.

This analysis is part of a feasibility study of breast cancer among women in South Carolina, the first to compare three ethnically different groups: two AA ethnic groups, those without known SI ancestry (non-SIs) and those considered SIs; and EAs. For simplicity and to recognize the importance of investigating breast cancer disparities between AAs and EAs, we use the term “race/ethnicity” as we explore the differences and similarities among these three groups.

Recognizing the implications for alcohol and tobacco use on survivorship, the primary purpose of this analysis was to characterize alcohol and tobacco use among women recently diagnosed with breast cancer across these three South Carolina racial/ethnic groups. Secondarily, we investigated whether patterns of use correlated with age or education (socioeconomic status and income were not available) and whether use within 6–21 months after diagnosis, as a surrogate for the years prior to diagnosis, correlated with cancer stage or tumor markers. We also compared alcohol consumption and cigarette smoking among our study sample with use in the general female population of South Carolina, of the same races (EA and AA) and age range as the study sample.

## Methods

### Human participant protection

This study was carried out in accordance with the US Department of Health and Human Subjects Policy for the Protection of Human Research Subjects. The protocol was approved by the IRBs of the Medical University of South Carolina (MUSC), and the South Carolina Department of Health and Environmental Control. All subjects gave verbal consent as allowed under the policies of the above IRBs, as the research presented no more than minimal risk of harm to subjects and involved no procedures for which written consent is normally required outside of the research context.

### Study design, case ascertainment, and recruitment

**Figure 1:**
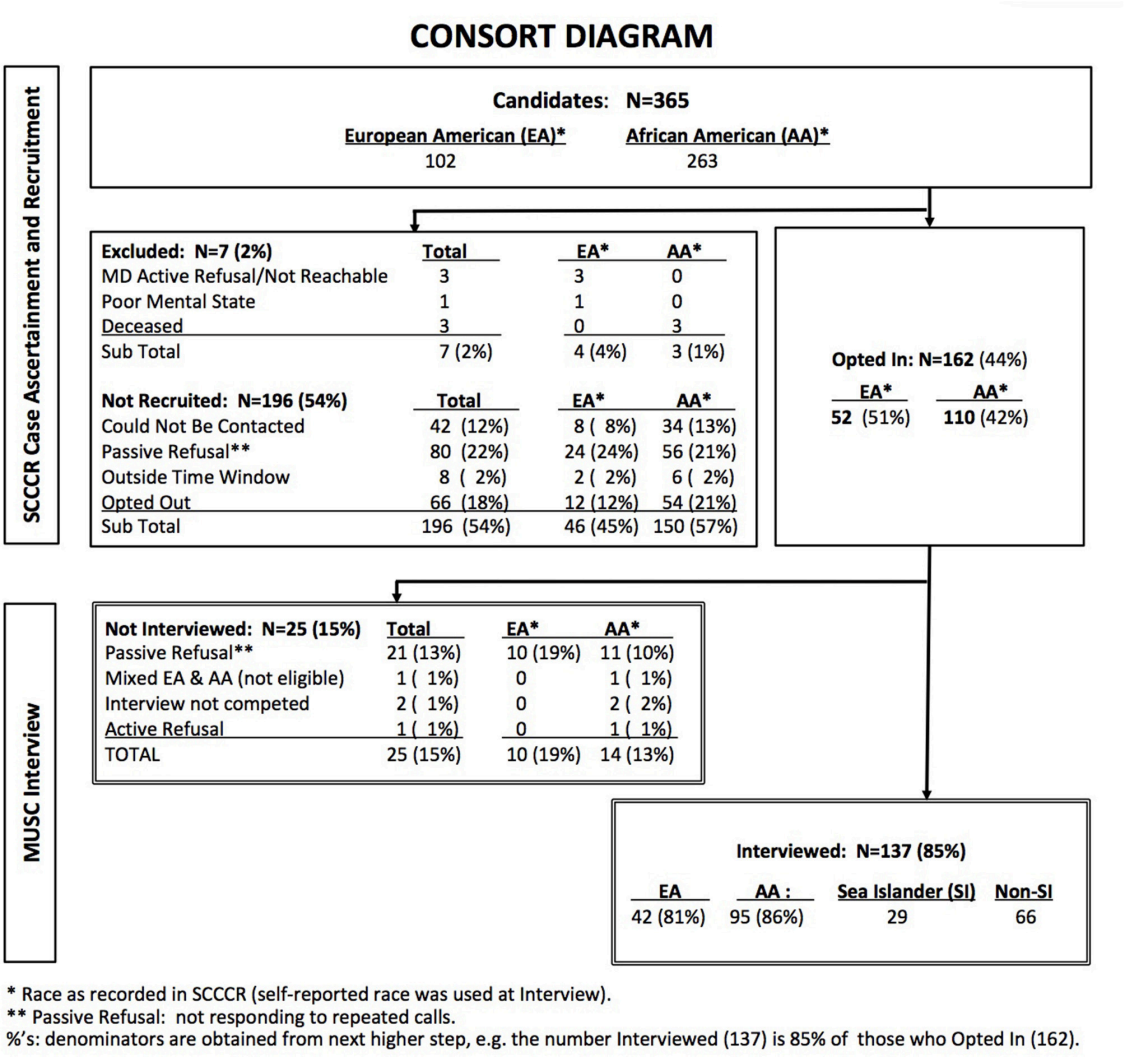
CONSORT diagram.

Case ascertainment and data collection steps in this cross-sectional study are illustrated in the CONSORT diagram (Figure 1). Potential study participants were identified through the South Carolina Central Cancer Registry (SCCCR) of the South Carolina Department of Health and Environmental Control. They consisted of adult women residing in South Carolina, diagnosed with invasive breast cancer of known stage at age 21 years or older, and whose race was recorded in the SCCCR as non-Hispanic, either Black (AA) or White (EA).

SCCCR study staff contacted each identified potentially eligible case by telephone after receiving passive physician approval [treating physician was asked to respond to investigators only to forbid contact with a potential subject (41)]. Women who declared an interest in participating (“opted in”) were referred to MUSC study personnel, who conducted a brief telephone interview. Cases were diagnosed between August 2011 and December 2012. All interviews were completed by December 2013, within 6–21 months of diagnosis. For this feasibility study, target sample size was 30 women in each of the three ethnic groups. This resulted in oversampling of AAs, as SI ethnicity was determined during MUSC interview.

### Ethnicity determination

Race/ethnicity of each participant was categorized based on that of parents and grandparents as reported in the MUSC interview: EA if the candidate considered all four grandparents to be of European origin, or AA if all four grandparents were AA. Among AAs, the study candidate was sub-classified as Sea Islander (SI) if (a) she considered herself to be a SI, coming from the SI geographic region of South Carolina (i.e., 30 miles or less from the Atlantic coast), and (b) either all four AA grandparents were born in this region or, in cases where the birthplace of all four grandparents was not known, at least both AA parents were born in this region. Women who did not meet these definitions, for example women of mixed race or unknown ancestry, or who reported Asian or Hispanic ancestry, were excluded from the study. Thus, this study included only Non-Hispanic EA and AA women.

The geographic definition of the SI region was established by researchers of the Sea Islander/Gullah people (38, 39), and recognizes the unique history and geography of the South Carolina coastal barrier (sea) islands region (40).

### Data collection

The SCCCR provided data on age at diagnosis, race and breast cancer characteristics (stage, tumor markers). Trained MUSC interviewers administered a short telephone interview with each participant to verify date of birth and obtain self-reported race, ethnicity, education, height and weight, plus alcohol consumption and cigarette smoking during the past 30 days.

Breast cancer estrogen receptor (ER) and progesterone receptor (PR) expression were categorized as “negative” if reported in the SCCCR data as “negative” or “borderline.” HER-2 expression was categorized as negative if reported as “negative,” “borderline,” or “within normal limits.” Tumors negative for all three markers were labeled triple-negative.

**Figure 2:**
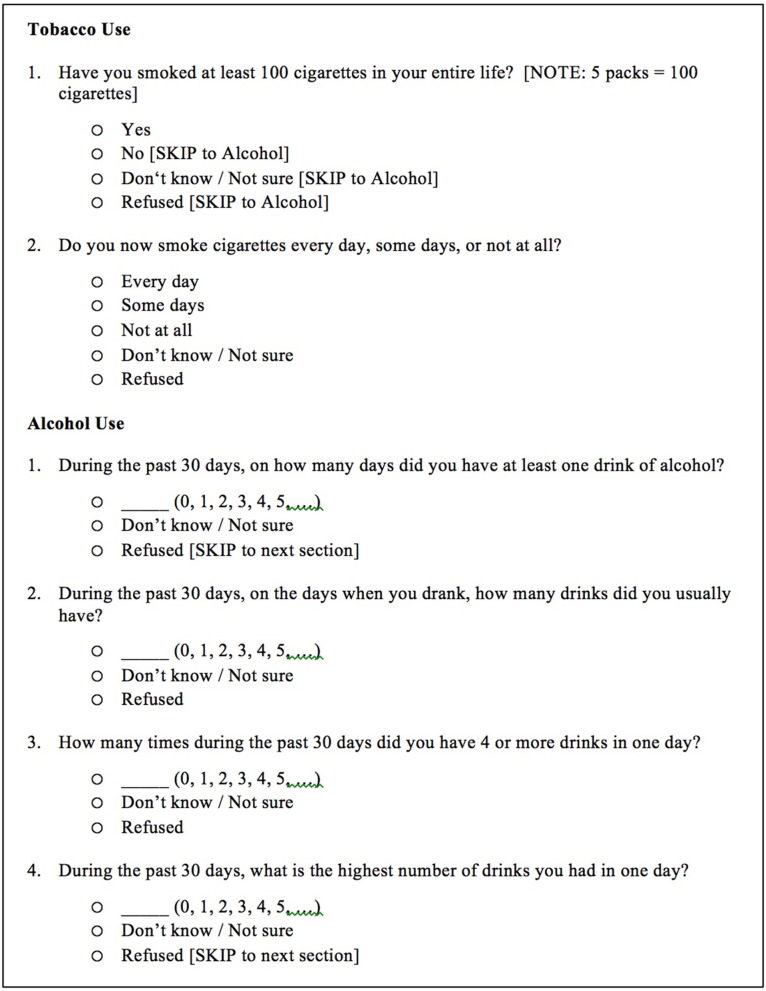
Questions used to determine tobacco and alcohol use. Questions were selected from the Tobacco Use and Alcohol Consumption modules of the Centers for Disease Control and Prevention (CDC) 2010 Behavioral Risk Factor Surveillance Survey (BRFSS). CDC (42).

Alcohol consumption and cigarette smoking during the past 30 days were ascertained using questions selected from the Centers for Disease Control and Prevention (CDC) 2010 Behavioral Risk Factor Surveillance Survey (BRFSS) (Figure 2) (42). The BRFSS is a state-based system of health surveys that provides valid and reliable estimates of population health risk behaviors, clinical preventive health practices, and health-care access, primarily related to chronic disease and injury (43, 44).

Published CDC guidelines were used to characterize alcohol and tobacco use (45, 46), where moderate alcohol consumption is defined as up to one drink/day (for women) consumed on any given day and not an average over several days, heavy consumption as a daily average of more than one drink/day (8 drinks or more per week), and binge drinking as four or more drinks on a single occasion. Because our questionnaire did not define “one drink” in terms of ounces consumed, we used the number of drinks as reported by the participants.

Tobacco use (cigarettes) was defined as “yes” if the participant reported having smoked at least 100 cigarettes during her lifetime (46). Smokers were categorized as “daily” if they reported now (time of interview) smoking cigarettes every day, “sometimes” if smoking on some days, and “none” if they reported not smoking now at all.

To assess how representative our study population was of incident breast cancer cases statewide, age at diagnosis and disease stage were compared to SCCCR data for all invasive cases (of known stage) diagnosed during 2011–2012 among non-Hispanic EA and AA women of the same age range as study participants. In addition, educational attainment, alcohol and tobacco use among study participants were compared with 2012–2013 South Carolina BRFSS data for women of the same age range as the study participants (provided by the Division of Surveillance of South Carolina Department of Health and Environmental Control). These data represent the entire female population, and are not restricted to women with breast cancer. Note that AA ethnicity is not available in BRFSS data.

### Statistical analysis

Participant demographic and tumor characteristics, alcohol and tobacco use were summarized using standard descriptive statistics. In accordance with the primary purpose of this study, statistical analyses examined differences among and between the three racial/ethnic groups: EAs, non-SI AAs, and SI AAs. Differences were evaluated using *t*-tests, Wilcoxon rank sum and Kruskal-Wallis H non-parametric analysis for continuous data as appropriate, and chi-square or Fisher exact tests for categorical data. Simple linear and logistic regression modeling techniques were used to investigate associations between alcohol consumption, tobacco use, age, and cancer characteristics. Age at interview was used in analyses of self-reported data. For comparison of breast cancer characteristics with population-based state-level data, age at diagnosis was used. Statistical analyses were conducted using STATA 10.1 (47). All tests were two-tailed; *p*-values less than 0.05 were considered statistically significant.

## Results

### Study population

The analytic study sample was derived from 365 potential candidates identified from the SCCCR by registry staff (CONSORT Diagram: Figure 1), of whom 162 (44%) opted-in and were referred to MUSC data collection personnel. Of these, 137 (85%) women met all eligibility criteria and completed the telephone interview. The study sample was thus comprised of 42 EAs, 66 non-SI AAs and 29 SIs. Recruitment rates did not differ significantly by race: 42% of AAs and 51% of EAs identified by SCCCR staff opted-in (*p* = 0.11), and 86 and 81% respectively of these completed the MUSC interview (*p* = 0.36).

Ascertainment of SIs presented specific challenges, as AA ethnicity is not recorded in the SCCCR. We therefore oversampled AAs, focusing on the coastal region, until reaching the target sample size of 30 participants per racial/ethnic group. This resulted in the non-SI AA sample (*n* = 66) exceeding our target. One SI was later determined to be ineligible, resulting in 29 SIs in the final analytic sample. EA telephone interviews were continued until SI recruitment was complete (*n* = 42).

### Socio-demographic characteristics

Age at interview ranged from 38.2 to 90.7 years (Table 1). EAs tended to be older (median age 63.0 years) than non-SI AAs (55.7) with SI's intermediate (58.6). Educational attainment, a surrogate for socio-economic status, did not vary significantly among the three patient groups, with 45% (SIs) to 55% (non-SI AAs) to 64% (EAs) having earned more than a highschool diploma.

**Table 1:** Participant demographics and breast cancer characteristics.

	**European American (EA)**		**African American (AA)**				***p*****-values**				
			**Non-Sea Islander**		**Sea Islander (SI)**		**EA vs. Non-SI vs. SI**	**EA vs. Non-SI**	**Non-SI vs. SI**	**SI vs. EA**	**EA vs. AA**
	**(*****N*** = **42)**		**(*****N*** = **66)**		**(*****N*** = **29)**						
	***N***	**%**	***N***	**%**	***N***	**%**					
**AGE AT INTERVIEW (YEARS)**											
Mean (std dev)	62.6	(±12.9)	57.6	(±12.0)	61.3	(±10.5)	0.091	0.044	0.160	0.648	0.086
Median (range)	63.0	(38.9–87.4)	55.7	(38.2–90.2)	58.6	(46.1–82.2)	0.107	0.053	0.160	0.607	0.101
**EDUCATION**											
< HS Diploma	3	7%	12	18%	8	28%	0.190	0.562	0.292	0.029	0.194
HS Diploma/12 yrs	12	29%	18	27%	8	28%					
Trade/Some College	16	38%	19	29%	3	10%					
College Degree	6	14%	10	15%	7	24%					
Post Grad Degree	5	12%	7	11%	3	10%					
>HS Diploma vs Other	27	64%	36	55%	13	45%	0.263	0.317	0.383	0.104	0.168
**BREAST CANCER**											
Localized	33	79%	31	47%	21	72%	0.011	0.004	0.074	0.886	0.027
Regional	8	19%	28	42%	7	24%					
Distant	1	2%	7	11%	1	3%					
Regional/Distant vs Localized	9	21%	35	53%	8	28%	0.002	0.001	0.026	0.582	0.008
ER Positive	38 of 42	90.5%	43 of 65	66.2%	24 of 29	82.8%	0.011	0.005	0.139	0.471	0.015
PR Positive	34 of 42	81.0%	38 of 65	58.5%	26 of 29	89.7%	0.002	0.020	0.004	0.506	0.150
Triple Negative	3 of 39	7.7%	14 of 61	23.0%	2 of 21	9.5%	0.102	0.058	0.220	1.000	0.114

*EA, European American; ER, estrogen receptor expression; HS, highschool; Non-SI, Non Sea Islander; PR, progesterone receptor expression; SI, Sea Islander; std dev, standard deviation*.

### Breast cancer characteristics

Non-SI AAs were most likely to be diagnosed with late stage disease (Regional or Distant), significantly different from EAs and SIs; and least likely to have estrogen receptor expression (ER) positive cancer, significantly different from EAs only. Among SIs, cancer stage, ER, PR and triple-negative status (negative for ER, PR, and HER-2 expression) were more similar to EAs than non-SI AAs, an unexpected finding (Table 1).

### Alcohol consumption

A minority of participants self-identified as consuming alcohol (Table 2), a practice more prevalent among EAs (40%) than either AA group (24% of non-SIs and 21% of SIs). Moderate drinking (defined as usual consumption of up to one drink per day on days alcohol is consumed) was also more prevalent among EAs (29%) than AAs (15 and 10% respectively). While no statistically significant differences were observed in 3-way tests, EAs were significantly more likely than AAs (non-SI and SI combined) to consume alcohol, and to practice moderate drinking (*p*-values each 0.038). About 9% (non-SIs) to 12% (EAs) consumed more than one glass per day when drinking, not different among or between racial/ethnic groups. Heavy drinking (more than one drink per day on average during the past 30 days) was rare, reported by only two of the 137 study participants. Binge drinking (consuming four or more drinks on any given day) was reported by 10% of both EAs and SIs, and 3% of non-SI AAs. Neither heavy nor binge drinking differed significantly among or between ethnic or racial groups.

**Table 2:** Alcohol consumption and cigarette smoking.

	**European American (EA)**		**African American (AA)**				***p*****-values**				
	**(*****N*** = **42)**		**Non-Sea Islander(*****N*** = **66)**		**Sea Islander (SI) (*****N*** = **29)**		**EA vs. Non-SI vs. SI**	**EA vs. non-SI**	**Non-SI vs. SI**	**SI vs. EA**	**EA vs. AA**
	***N***	**%**	***N***	**%**	***N***	**%**					
**ALCOHOL USE (LAST 30 DAYS)**											
Consumers (vs. non-consumers)	17	40.5%	16	24.2%	6	20.7%	0.125	0.074	0.796	0.121	0.038
**Usual Drinks/Day (On Days Consumed)^a^**											
None	25	59.5%	50	75.8%	23	79.3%	0.281	0.158	0.860	0.145	0.086
Moderate (≤ 1)	12	28.6%	10	15.2%	3	10.3%					
>1	5	11.9%	6	9.1%	3	10.3%					
Moderate (≤ 1 drink on days consumed)^a^	12	28.6%	10	15.2%	3	10.3%	0.105	0.091	0.748	0.081	0.038
Heavy (average >1 drink/day)^b^	1	2.4%	0	0.0%	1	3.5%	0.267	0.389	0.305	1.000	0.521
Binge drinking (≥4 drinks any day)	4	9.5%	2	3.0%	3	10.3%	0.210	0.205	0.164	1.000	0.456
**Days/Month^c^**											
None	25	59.5%	50	75.8%	23	79.3%	0.239	0.133	0.705	0.259	0.077
1-7	11	26.2%	13	19.7%	4	13.8%					
10-30	6	14.3%	3	4.6%	2	6.9%					
**Age At Interview (Years)**											
Consumers (N = 39)	17		16		6						
Mean (std dev)		54.7 (± 8.6)		51.0 (± 10.8)		57.1 (± 11.6)	–	–	–	–	–
Median (range)		52.3 (38.9-68.0)		49.4 (38.2–72.3)		53.9 (47.2–78.6)	0.356	0.234	0.210	1.000	0.350
Non-consumers (N = 98)	25		50		23						
Mean (std dev)		68.0 (± 12.7)		59.7 (± 11.7)		62.4 (± 10.2)	–	–	–	–	–
Median (range)		71.0 (40.2–87.4)		61.1 (39.0–90.2)		58.9 (46.1–82.2)	0.017	0.005	0.348	0.089	0.007
Consumers vs Non-consumers^d^		p = 0.001		p = 0.012		p = 0.258					
**CIGARETTE SMOKING**											
Ever Smoked vs Never	21	50.0%	19	28.8%	7	24.1%	0.040	0.026	0.804	0.047	0.010
Current: Daily/Occasionally vs None	6	14.3%	9	13.6%	2	6.9%	0.704	1.000	0.494	0.458	0.779
Never / Quit	36	85.7%	57	86.4%	27	93.1%	0.354	0.164	0.763	0.489	0.182
Occasionally	1	2.4%	6	9.1%	1	3.4%					
Daily	5	11.9%	3	4.5%	1	3.4%					
**ALCOHOL USE AND/OR CURRENT SMOKING**											
Neither	21	50.0%	45	68.2%	22	75.9%	0.318	0.192	0.922	0.178	0.083
Alcohol only	15	35.7%	12	18.2%	5	17.2%					
Cigarette smoking only	4	9.5%	5	7.6%	1	3.4%					
Alcohol and cigarettes	2	4.8%	4	6.1%	1	3.4%					
Alcohol and/or cigarettes	21	50.0%	21	31.8%	7	24.1%	0.065	0.070	0.626	0.047	0.033

*SI, Sea Islander; std dev, standard deviation*.

a *Usual consumption on days when alcohol was consumed*.

b *Average drinks/day over past 30 days exceeds 1 drink*.

c *No participants consumed alcohol on 8–9 days per month*.

d *Using non-parametric test of median ages*.

Alcohol consumers tended to be younger than abstainers (Tables 2, 3), regardless of race/ethnicity. Compared to abstainers, median age of consumers was 19 years younger among EAs (*p* < 0.001), 11 years younger among non-SI AAs (*p* = 0.010), and 5 years younger among SIs (*p* = 0.283). Alcohol use was two to three times more prevalent among women with a college degree, a non-significant trend seen in EAs and non-SI AAs but not among SIs (Table 3).

**Table 3:** Alcohol consumption and cigarette smoking: associations with patient demographics and cancer characteristics.

	**Population**	***N***	**Odds ratio**	***p*-value**	**95% Confidence interval**
**ALCOHOL CONSUMPTION (YES vs. NO)**					
Age at Interview (year)	All^a^	137	0.92	< 0.001	0.88; 0.96
	EA	42	0.90	0.003	0.84; 0.97
	AA: non-Sea Islander	66	0.93	0.015	0.88; 0.99
	AA: Sea Islander	29	0.95	0.280	0.86; 1.05
Education: College Degree^b^	All^a^	137	2.05	0.110	0.85; 4.93
	EA	42	2.35	0.294	0.48; 11.54
	AA: non-Sea Islander	66	3.02	0.091	0.84; 10.86
	AA: Sea Islander	29	0.71	0.738	0.10; 5.26
Cancer Stage: Regional/Distant vs. Localized ^b^	All^a^	137	3.29	0.013	1.29; 8.38
	EA	42	0.32	0.280	0.04; 2.55
	AA: non-Sea Islander	66	6.35	0.014	1.47; 27.56
	AA: Sea Islander	29	9.33	0.034	1.19; 73.16
**CURRENTLY SMOKE CIGARETTES (DAILY OR OCCASIONALLY)**					
Age at Interview (year)	All^a^	137	0.96	0.058	0.91; 1.00
	EA	42	0.95	0.196	0.88; 1.03
	AA: non-Sea Islander	66	0.96	0.213	0.90; 1.02
	AA: Sea Islander	29	0.95	0.523	0.81; 1.12
Education: Some college or	All^a^	137	0.19	0.010	0.05; 0.67
Greater^b^^,^ ^c^	EA	42	0.13	0.055	0.02; 1.05
	AA: non-Sea Islander	66	0.30	0.153	0.06; 1.57
	AA: Sea Islander	29	–^(d)^	–	–

*AA, African American; EA, European American*.

a *Adjusted for race/ethnicity*.

b *Adjusted for age at interview*.

c *Attainment of a college degree was rare among smokers, therefore “Some college or greater” was used*.

d *Data inadequate for logistic analysis, due to collinearity*.

Late stage disease (Regional or Distant) was significantly associated with consuming alcohol (Table 3), specifically among AAs whether SI (odds ratio (OR) = 6.0, *p* = 0.015) or non-SI (OR = 9.5, *p* = 0.033). However 95% confidence intervals were very wide even with all AAs combined, reflecting small cells, and so these findings should be interpreted with caution. Alcohol consumption was not consistently or significantly associated with tumor ER, PR, or triple-negative status in any racial/ethnic group (data not shown).

### Cigarette smoking

Ever smoking (Table 2) was reported by 50% of EAs, almost twice that of the AA groups (29% of non-SI AAs, *p* = 0.026 and 24% of SIs, *p* = 0.047). In contrast, only 12% of participants reported current use of cigarettes (daily or occasionally), with no significant differences by racial/ethnic group. As with alcohol consumption, current smoking was less common among older women of each race/ethnicity although not statistically significant (Table 3). Women with at least some college education were less likely to smoke (OR = 0.19; *p* = 0.010), a trend observed but not significant within each racial/ethnic group. No significant or consistent associations were observed between current smoking and cancer stage, or ER status.

### Alcohol consumption and/or cigarette smoking

Consuming alcohol and/or currently smoking cigarettes was more prevalent among EAs (50%) than non-SI AAs (32%, *p* = 0.070) or SIs (24%, *p* = 0.047), reflecting the higher use of alcohol than cigarettes. One third to one half of current smokers (EAs 33%, non-SI AAs 44%, and SIs 50%) also consumed alcohol, but very few participants reported this double exposure (3–6% of each racial/ethnic group).

### Comparison with state-level population-based data

To see how well our study sample represented the population of breast cancer cases from which they were drawn, we compared our study sample with all statewide cases on several parameters (Table 4). As with the study sample, this comparison was restricted to invasive breast cancer of known stage diagnosed in non-Hispanic White and Black women in the same age range as our sample during the study period (2011–2012). Mean ages at diagnosis were very similar: EAs in our sample were less than two than years younger than all South Carolina cases. However, our EA sample included fewer cases of later stage (Regional or Distant) than in the SCCCR (21 vs. 35%). Of interest, among state-level AA cases (SI ethnicity data are not available in the SCCCR) mean age at diagnosis and prevalence of later stage disease were between the corresponding values found in our non-SI and SI study samples.

**Table 4:** Demographics, cancer stage, alcohol consumption and cigarette smoking: study population compared to state-level data.

	**European American (White^a^^,^^b^)**		**African American (Black^a^^,^^b^)**		
	**Current study**	**SCCCR^a^, BRFSS^b^**	**Current study**		**SCCCR^a^, BRFSS^b^**
			**Non-SI**	**SI**	
	**(*N* = 42)**		**(*N* = 66)**	**(*N* = 29)**	
Mean age at Diagnosis (years)	61.6	63.3^a^	56.7	60.2	59.3^a^
Regional/Distant stage	21%	35%^a^	53%	28%	48%^a^
Education:					
More than Highschool	64%	57%^b^	55%	45%	44%^b^
College degree	26%	23%^b^	26%	34%	15%^b^
Alcohol Consumption (last 30 days):					
Yes	40%	41%^b^	24%	21%	28%^b^
Heavy drinkers (>1 drink per day)	2%	6%^b^	0%	4%	3%^b^
Binge drinking (4+ drinks any day)	10%	7%^b^	3%	10%	6%^b^
Cigarette Smoking:					
Ever smoked	50%	48%^b^	29%	24%	33%^b^
Current: Daily/Occasionally	14%	17%^b^	14%	7%	14%^b^

*BRFSS, Behavioral Risk Factor Surveillance Survey; SCCCR, South Carolina Central Cancer Registry; SI, Sea Islanders*.

a *SCCCR, Includes all cases of invasive breast cancer of known stage diagnosed throughout South Carolina in 2011–2012, among adult non-Hispanic White (N = 4,848) or Black (N = 1,580) women within the age range of study participants*.

b *BRFSS, Education, Alcohol consumption, Cigarette use: from South Carolina BRFSS 2012–2013, for non-Hispanic White and Black women within the age range of study participants, provided by the Division of Surveillance of SC DHEC*.

We also compared education, alcohol consumption and cigarette smoking among our study sample with South Carolina population-based BRFSS data for non-Hispanic White and Black women of the study sample age range. Our study cases were more likely to have greater than a highschool diploma, or a college degree, than in BRFSS data, particularly among EAs and non-SI AAs. Taken together, these results suggest some recruitment bias toward a better-educated study sample, with less advanced disease at least among EAs.

Prevalence of some alcohol consumption during the past 30 days was very similar among our EA study cases and the BRFSS general population, and somewhat lower among our AA cases. Heavy and binge drinking prevalence did not exceed 10% of study cases or 7% of the BRFSS general population. In both our study sample and BRFSS data, current smoking was much less prevalent than a history of ever smoking. These patterns were similar among EAs of both populations. In contrast, non-SI AAs resembled BRFSS AAs, while SIs were less likely to have ever smoked and half as likely to be current smokers compared to AAs in the BRFSS sample.

## Discussion

The purpose of this study was to evaluate the prevalence of modifiable outcome risk factors, alcohol and tobacco use, in a genetically diverse population of recently diagnosed breast cancer patients. Three groups were chosen as highly relevant to understanding breast cancer disparities in South Carolina, and as a representation of diversified genetics in the United States. This diverse sample consisted of European Americans, African Americans with varied genetic admixture, and African American Sea-Islanders who have remained culturally and geographically isolated, allowing for preservation of their African genetics (39) and potentially unique cultural and environmental influences (40). It is well established that AA's have higher mortality from invasive breast cancer when compared to their EA counterparts, despite the fact that they are less likely to be diagnosed with this disease. For many years now, research by many investigators has focused on addressing and understanding why this survival disparity exists, strongly suggesting a multifactorial and complex interaction of tumor biology, stage at diagnosis, comorbidities, and environmental influences (48). In addition, geographic variation in mortality may be associated with social factors and access to health care (49–51). While factors such as tumor biology and genetics cannot be modified, life-style factors can potentially be modified through behavioral adjustments.

### Alcohol

Alcohol consumption after breast cancer diagnosis may modestly increase risk of recurrence, particularly in post-menopausal women, as well as breast cancer mortality and all-cause mortality (3, 4), although this remains controversial with conflicting reports, possibly influenced by differences in study design and analyses (5–7, 52, 53). Alcohol is also a known breast cancer risk factor (54–56), with the risk of invasive breast cancer increased with greater daily alcohol consumption and likely greatest for hormone sensitive cancers (57). These observations may result from the inherent carcinogenic effects of alcohol through its metabolism to acetaldehyde (16–19).

Among our sample of women recently diagnosed with breast cancer, EAs were almost twice as likely as AAs (*p* = 0.038), regardless of SI ancestry, to report consuming alcohol in the past 30 days: 40% of EAs, 24% of non-SIs and 21% of SIs. Moderate consumption (up to one drink per day on days when alcohol was consumed) was also significantly higher among EAs than AAs, comprising one-half to one-third of drinkers respectively, while about 10% of each group consumed more than one drink per day on such days. Similar racial differences have also been found in other work, including the Carolina Breast Cancer Study of breast cancer survivors in North Carolina (44), with AA's reporting less alcohol consumption than EA's (16, 58–60). This difference may be driven by social customs, with AA women more likely than EAs to choose not to drink for religious or cultural reasons (61).

Multiple studies have evaluated timing of alcohol exposure and age, and it is likely that exposure to alcohol at early ages may also affect a woman's lifetime risk of developing breast cancer (20, 62). Specifically, nulliparous breast tissue seems to be more susceptible to neoplastic transformation and thus the carcinogenic effect of alcohol in adolescent and early adult years may contribute to cancer development (63–65). This is of particular concern as younger women are increasingly likely to participate in binge drinking, and greater consumption and binge drinking may increase breast cancer risk (66). In our study (cases ranged in age from 37 to 89 years) drinkers were, on average, younger than those who consumed no alcohol, with few consumers over age 70. Moderate intake was more common than binge drinking, which ranged from 0 to 10% (although half of SIs who consumed alcohol reported binge-drinking). While we do not know whether study participants had reduced their consumption since breast diagnosis, or when they began consuming alcohol, it seems unlikely that women increased their intake post diagnosis.

In our study, AAs were more likely than EAs to be diagnosed with later stage cancer and to have ER or PR negative disease. The more aggressive and less prognostically favorable breast cancer subtype among AAs is a well-established racial disparity. We found later stage disease significantly associated with alcohol consumption (but not with amount consumed or binge drinking) among AAs only, regardless of SI ethnicity. While these associations have wide confidence intervals, this observation suggests that alcohol consumption may bear further investigation as a contributor to racial disparities in breast cancer risk.

### Tobacco

Smoking is associated with a 2-fold higher rate of dying from breast cancer compared to never smoking (24), as well as factors leading to poorer outcomes among women with breast cancer: including lower socioeconomic status (31), decreased physical activity (67), and comorbidities (34). Nechuta et al. (53) evaluated the late effects of post-diagnosis lifestyle factors in a prospective sample of 6,295 ER positive Stage I-III breast cancer survivors, for whom risk of late recurrence was of concern, in three pooled cohorts from Shanghai, China (one study) and the US (2 studies). Former heavy and current smokers had approximately 30% increased risks of late recurrence, compared to never smokers, as well as increased breast cancer-specific and all-cause mortality, however it is not clear whether results were adjusted for alcohol consumption.

In our study, 14% of EAs, 14% of non-SIs and 7% of SIs (no significant differences) reported current smoking (occasionally or daily). This is somewhat lower than reported for women in North Carolina with recently diagnosed breast cancer (22% of EAs and AAs), but consistent with their finding of no racial difference in current smoking (25). In our study sample, EA's were almost twice as likely to have ever smoked compared to the AA groups, suggesting a greater degree of experimentation earlier in life or that many women had quit. However, we do not know whether prior smokers had quit smoking earlier in life, or subsequent to their breast cancer diagnosis. Current smoking was more prevalent among younger women and those with less education, consistent with the Carolina Breast Cancer Study (25). One third to one half of current smokers also consumed alcohol, a double exposure that was nonetheless rare and reported by only seven participants (3–6% by ethnicity), too infrequent to investigate racial/ethnic differences.

In the last few years through large studies and meta-analyses, cigarette smoking, whether active or passive, has emerged as conferring moderate risk for breast cancer (68, 69), the effect confounded by the effects of alcohol consumption (36, 57). Multiple studies suggest a correlation between smoking and alcohol use, and recent research shows that these two behavioral effects combined may have deleterious effects not only on breast cancer risk but also on survivorship.

### Strengths and limitations

Our study is the first to use a statewide sampling approach to investigate alcohol use and cigarette smoking among women of South Carolina with recently diagnosed breast cancer, and to specifically include Sea Islanders. Based upon comparison with all SCCCR breast cancer cases, our EA study sample included fewer cases with late stage disease than expected, suggesting a possible EA recruitment bias toward early stage disease; this pattern was not seen among AAs combined as a single group. However, with no similar studies of SIs for comparison and SI ethnicity not recorded in the SCCCR, we cannot know whether our study sample reflects age and disease characteristic typical of this AA subgroup.

As with any study relying on self-reported behavioral data, there are some limitations. There is potential for recall bias if respondents found it difficult to remember and estimate the number of alcoholic drinks consumed in the past 30 days, and number of days involved. Some women may also have under-reported their consumption (social desirability bias). Our data for alcohol and tobacco use were obtained at between 6 and 21 months of diagnosis. We do not know whether or how these behaviors may have changed since diagnosis or may change again while women continue treatment and enter the “survivor” phase. To the extent that alcohol use may impact breast cancer stage at diagnosis and/or subsequent recurrence and survival, if prevalence and level of consumption were under-reported our data under-estimate the seriousness of our findings.

This is the first study to investigate breast cancer among the Sea Islanders, a unique African American ethnic group, estimating differences in alcohol and tobacco utilization among three racial/ethnic groups in South Carolina. It is an exploratory feasibility study, obtaining cases from the SCCCR, rather than a hospital-based design, in order to obtain a state-wide representative sample of patients including SI patients. It provides a basis for further investigations among AA ethnic groups, toward better understanding of racial and ethnic disparities in breast cancer (and possibly other) outcomes. Differences and similarities between and across racial/ethnic groups are presented as a first estimate, not as a definitive statement, and confidence intervals reflect sample sizes. We acknowledge that sample sizes of EAs, and particularly SIs, were relatively small in comparison to the number of non-SI AAs. However, that statistically significant differences were found even with these relatively small sample sizes suggests that differences in breast cancer characteristics and patterns of alcohol and cigarette use between AA subgroups do bear additional scrutiny.

## Conclusions

We have described patterns of alcohol and cigarette use among recently diagnosed invasive breast cancer cases belonging to the three major racial/ethnic groups of South Carolina: EAs, and AAs with and without Sea Island ancestry.

In our statewide sample we found that race-specific patterns of alcohol consumption and cigarette use were similar to or perhaps lower than those in the general South Carolina population, with EAs more likely to consume alcohol than AAs. However, the association between tumor stage and alcohol use, among AAs, is suggestive and differential effects of these exposures on breast cancer risk as well as survival among the racial/ethnic groups cannot be ruled out.

Although moderate alcohol use and frequency of consumption were greater among EAs than AAs, heavier consumption and binge drinking were similar among all groups. In general, alcohol consumption was more common among younger women, in whom breast cancer is often of poorer prognosis. Cigarette smoking was infrequent in every group, but 50% of EAs, 32% of non-SI AAs, and 24% of SIs used one or both products. Given the carcinogenic effects of alcohol, the deleterious health impacts of frequent moderate (or greater) alcohol consumption and of smoking, the known and potential impacts on cancer outcomes and survival, and the potential for differences in alcohol effects on either incidence or survival according to genetic heritage, we recommend that women (and men) with breast cancer be educated and actively assisted in reducing or eliminating these exposures.

## Author contributions

AA and RK: co-primary investigator, study development and management; CB and HV: participant interviews; CM: case identification and recruitment; DB, KK and TH: study and data management; DH: case identification and recruitment, cancer data; JC: study development, data analysis and interpretation, manuscript preparation; MF: primary investigator, study development and management, manuscript preparation; SB: cancer registry interface, case identification and recruitment, cancer data; VB: hypothesis development, participant interviews, manuscript preparation.

### Conflict of interest statement

The authors declare that the research was conducted in the absence of any commercial or financial relationships that could be construed as a potential conflict of interest. The reviewer DD and the handling Editor declared their shared affiliation.

## Abbreviations

AA: African-American
BC: Breast cancer
BRFSS: Behavior and Risk-Factor Surveillance System
CDC: Centers for Disease Control and Prevention
EA: European-American
ER: Estrogen receptor
MUSC: Medical University of South Carolina
OR: Odds ratio
PR: Progesterone receptor
SC: South Carolina
SCCCR: South Carolina Central Cancer Registry
SI: Sea Islander.

## References

[1] SEER Cancer Statistics Review, 1975-2013, National Cancer Institute. Available online at: https://seer.cancer.gov/csr/1975_2013/, based on November 2015 SEER data submission, posted to the SEER web site, April 2016

[2] SC DHEC South Carolina Department of Health and Environmental Control. SCAN Cancer Mortality Data. Available online at: http://scangis.dhec.sc.gov/scan/cancer2/mortinput.aspx (Accessed April 13, 2018).

[3] Holm M Olsen A Christensen J Kroman NT Bidstrup PE Johansen C. Pre-diagnostic alcohol consumption and breast cancer recurrence and mortality: results from a prospective cohort with a wide range of variation in alcohol intake. Int J Cancer (2013) 132:686–94. 10.1002/ijc.27652 22623182

[4] Kwan ML Chen WY Flatt SW Weltzien EK Nechuta SJ Poole EM. Postdiagnosis alcohol consumption and breast cancer prognosis in the after breast cancer pooling project. Cancer Epidemiol Biomarkers Prev. (2013) 22:32–41. 10.1158/1055-9965.EPI-12-1022 23150063 PMC3538884

[5] Flatt SW Thomson CA Gold EB Natarajan L Rock CL Al-Delaimy WK Low to moderate alcohol intake is not associated with increased mortality after breast cancer. Cancer Epidemiol Biomarkers Prev. (2010) 19:681–8. 10.1158/1055-9965.EPI-09-0927 20160253 PMC2836421

[6] Newcomb PA Kampman E Trentham-Dietz A Egan KM Titus LJ Baron JA. Alcohol consumption before and after breast cancer diagnosis: associations with survival from breast cancer, cardiovascular disease, and other causes. J Clin Oncol. (2013) 31:1939–46. 10.1200/JCO.2012.46.5765 23569314 PMC3661933

[7] McTiernan A Irwin M Vongruenigen V. Weight, physical activity, diet, and prognosis in breast and gynecologic cancers. J Clin Oncol. (2010) 28:4074–80. 10.1200/JCO.2010.27.9752 20644095 PMC2940425

[8] McDonald PA Williams R Dawkins F Adams-Campbell LL. Breast cancer survival in African American women: is alcohol consumption a prognostic indicator? Cancer Causes Control (2002) 13:543–9. 10.1023/A:1016337102256 12195644

[9] Li CI Chlebowski RT Freiberg M Johnson KC Kuller L Lane D. Alcohol consumption and risk of postmenopausal breast cancer by subtype: the women's health initiative observational study. J Natl Cancer Inst. (2010) 102:1422–31. 10.1093/jnci/djq316 20733117 PMC2943525

[10] Tjonneland A Christensen J Olsen A Stripp C Thomsen BL Overvad K. Alcohol intake and breast cancer risk: the European Prospective Investigation into Cancer and Nutrition (EPIC). Cancer Causes Control (2007) 18:361–73. 10.1007/s10552-006-0112-9 17364225

[11] Allen NE Beral V Casabonne D Kan SW Reeves GK Brown A. Moderate alcohol intake and cancer incidence in women. J Natl Cancer Inst. (2009) 101:296–305. 10.1093/jnci/djn514 19244173

[12] Guerra Guerrero V Fazzi Baez A Cofre Gonzalez CG Mino Gonzalez CG Monitoring modifiable risk factors for breast cancer: an obligation for health professionals. Rev Panam Salud Publica. (2017) 41:e80. 28614486 10.26633/RPSP.2017.80 PMC6645182

[13] Cao Y Willett WC Rimm EB Stampfer MJ Giovannucci EL. Light to moderate intake of alcohol, drinking patterns, and risk of cancer: results from two prospective US cohort studies. BMJ (2015) 351:h4238. 10.1136/bmj.h4238 26286216 PMC4540790

[14] Lew JQ Freedman ND Leitzmann MF Brinton LA Hoover RN Hollenbeck AR. Alcohol and risk of breast cancer by histologic type and hormone receptor status in postmenopausal women: the NIH-AARP Diet and Health Study. Am J Epidemiol. (2009) 170:308–17. 10.1093/aje/kwp120 19541857 PMC2727171

[15] Scoccianti C Lauby-Secretan B Bello PY Chajes V Romieu I. Female breast cancer and alcohol consumption: a review of the literature. Am J Prev Med. (2014) 46(3 Suppl. 1):S16–25. 10.1016/j.amepre.2013.10.031 24512927

[16] Volcik KA Ballantyne CM Fuchs FD Sharrett AR Boerwinkle E. Relationship of alcohol consumption and type of alcoholic beverage consumed with plasma lipid levels: differences between Whites and African Americans of the ARIC study. Ann Epidemiol. (2008) 18:101–7. 10.1016/j.annepidem.2007.07.103 17855114 PMC2819069

[17] Baan R Straif K Grosse Y Secretan B El Ghissassi F Bouvard V. Carcinogenicity of alcoholic beverages. Lancet Oncol. (2007) 8:292–3. 10.1016/S1470-2045(07)70099-2 17431955

[18] Helander A Lindahl-Kiessling K. Increased frequency of acetaldehyde-induced sister-chromatid exchanges in human lymphocytes treated with an aldehyde dehydrogenase inhibitor. Mutat Res. (1991) 264:103–7. 10.1016/0165-7992(91)90124-M 1944390

[19] Seitz HK Stickel F. Molecular mechanisms of alcohol-mediated carcinogenesis. Nat Rev Cancer (2007) 7:599–612. 10.1038/nrc2191 17646865

[20] Colditz GA Frazier AL. Models of breast cancer show that risk is set by events of early life: prevention efforts must shift focus. Cancer Epidemiol Biomarkers Prev. (1995) 4:567–71. 7549816

[21] Jung SY Papp JC Sobel EM Zhang ZF. Genetic variants in metabolic signaling pathways and their interaction with lifestyle factors on breast cancer risk: a random survival forest analysis. Cancer Prev Res. (2018) 11:44–51. 10.1158/1940-6207.CAPR-17-0143 29074537 PMC5754228

[22] Liu Y Nguyen N Colditz GA. Links between alcohol consumption and breast cancer: a look at the evidence. Womens Health (2015) 11:65–77. 10.2217/WHE.14.62 25581056 PMC4299758

[23] Candelaria NR Weldon R Muthusamy S Nguyen-Vu T Addanki S Yoffou PH. Alcohol regulates genes that are associated with response to endocrine therapy and attenuates the actions of tamoxifen in breast cancer cells. PLoS ONE (2015) 10:e0145061. 10.1371/journal.pone.0145061 26661278 PMC4681367

[24] Braithwaite D Izano M Moore DH Kwan ML Tammemagi MC Hiatt RA. Smoking and survival after breast cancer diagnosis: a prospective observational study and systematic review. Breast Cancer Res Treat (2012) 136:521–33. 10.1007/s10549-012-2276-1 23053660 PMC3507472

[25] Parada H Jr Sun X Tse CK Olshan AF Troester MA Conway K. Active smoking and survival following breast cancer among African American and non-African American women in the Carolina Breast Cancer Study. Cancer Causes Control (2017) 28:929–38. 10.1007/s10552-017-0923-x 28695396 PMC5709174

[26] Daniell HW. Increased lymph node metastases at mastectomy for breast cancer associated with host obesity, cigarette smoking, age, and large tumor size. Cancer (1988) 62:429–35. 10.1002/1097-0142(19880715)62:2<429::AID-CNCR2820620230>3.0.CO;2-4 3383142

[27] Murin S Inciardi J. Cigarette smoking and the risk of pulmonary metastasis from breast cancer. Chest (2001) 119:1635–40. 10.1378/chest.119.6.1635 11399684

[28] Scanlon EF Suh O Murthy SM Mettlin C Reid SE Cummings KM. Influence of smoking on the development of lung metastases from breast cancer. Cancer (1995) 75:2693–9. 10.1002/1097-0142(19950601)75:11<2693::AID-CNCR2820751109>3.0.CO;2-E 7743472

[29] Kobrinsky NL Klug MG Hokanson PJ Sjolander DE Burd L. Impact of smoking on cancer stage at diagnosis. J Clin Oncol. (2003) 21:907–13. 10.1200/JCO.2003.05.110 12610192

[30] Holmes MD Murin S Chen WY Kroenke CH Spiegelman D Colditz GA. Smoking and survival after breast cancer diagnosis. Int J Cancer (2007) 120:2672–77. 10.1002/ijc.22575 17278091

[31] Franzini L Williams AF Franklin J Singletary SE Theriault RL. Effects of race and socioeconomic status on survival of 1,332 black, Hispanic, and white women with breast cancer. Ann Surg Oncol. (1997) 4:111–8. 10.1007/BF02303792 9084846

[32] Sternfeld B Weltzien E Quesenberry CP Jr Castillo AL Kwan M Slattery ML. Physical activity and risk of recurrence and mortality in breast cancer survivors: findings from the LACE study. Cancer Epidemiol Biomarkers Prev. (2009) 18:87–95. 10.1158/1055-9965.EPI-08-0595 19124485 PMC3507507

[33] Tammemagi CM Nerenz D Neslund-Dudas C Feldkamp C Nathanson D. Comorbidity and survival disparities among black and white patients with breast cancer. JAMA (2005) 294:1765–72. 10.1001/jama.294.14.1765 16219879

[34] Satariano WA Ragland DR. The effect of comorbidity on 3-year survival of women with primary breast cancer. Ann Intern Med. (1994) 120:104–10. 10.7326/0003-4819-120-2-199401150-00002 8256968

[35] Slattery ML Curtin K Giuliano AR Sweeney C Baumgartner R Edwards S. Active and passive smoking, IL6, ESR1, and breast cancer risk. Breast Cancer Res Treat. (2008) 109:101–11. 10.1007/s10549-007-9629-1 17594514 PMC2532584

[36] Jones ME Schoemaker MJ Wright LB Ashworth A Swerdlow AJ. Smoking and risk of breast cancer in the Generations Study cohort. Breast Cancer Res. (2017) 19:118. 10.1186/s13058-017-0908-4 29162146 PMC5698948

[37] Butler EN Tse CK Bell ME Conway K Olshan AF Troester MA. Active smoking and risk of luminal and basal-like breast cancer subtypes in the Carolina Breast Cancer Study. Cancer Causes Control (2016) 27:775–86. 10.1007/s10552-016-0754-1 27153846 PMC5030064

[38] McLean DC Jr Spruill I Argyropoulos G Page GP Shriver MD Garvey WT. Mitochondrial DNA (mtDNA) haplotypes reveal maternal population genetic affinities of Sea Island Gullah-speaking African Americans. Am J Phys Anthropol. (2005) 127:427–38. 10.1002/ajpa.20047 15624208

[39] Parra EJ Kittles RA Argyropoulos G Pfaff CL Hiester K Bonilla C. Ancestral proportions and admixture dynamics in geographically defined African Americans living in South Carolina. Am J Phys Anthropol. (2001) 114:18–29. 10.1002/1096-8644(200101)114:1<18::AID-AJPA1002>3.0.CO;2-2 11150049

[40] Jackson J Slaughter S Blake HJ The Sea Islands as a cultural resource. Black Scholar. (1974) 5:32–9. 10.1080/00064246.1974.11431390

[41] Gurwitz JH Guadagnoli E Landrum MB Silliman RA Wolf R Weeks JC. The treating physician as active gatekeeper in the recruitment of research subjects. Med Care (2001) 39:1339–44. 10.1097/00005650-200112000-00009 11717575

[42] Centers for Disease Control and Prevention (CDC) Behavioral Risk Factor Surveillance System 2010 Questionnaire (2009). Available online at: https://www.cdc.gov/brfss/questionnaires/pdf-ques/2010brfss.pdf (Accessed April 13, 2018).

[43] Mokdad AH Stroup DF Giles WH. Behavioral Risk Factor Surveillance Team. Public health surveillance for behavioral risk factors in a changing environment. Recommedations from the Behavioral Risk Factor Surveillance Team. MMWR Recomm Rep. (2003) 52:1–12. 12817947

[44] Nelson DE Holtzman D Bolen J Stanwyck CA Mack KA. Reliability and validity of measures from the Behavioral Risk Factor Surveillance System (BRFSS). Soz Praventivmed. (2001) 46(Suppl. 1):S3–42. 11851091

[45] CDC/National Center for Health Statistics National Health Interview Survey. Glossary-Alcohol. Available online at: https://www.cdc.gov/nchs/nhis/alcohol/alcohol_glossary.htm (Accessed April 13, 2018).

[46] CDC/National, Center for Health Statistics National Health Interview Survey. Available online at: https://www.cdc.gov/nchs/nhis/tobacco/tobacco_glossary.htm (Accessed April 13, 2018).

[47] StataCorp Stata Statistical Software: Release 10. College Station, TX: StataCorp LP (2007).

[48] Daly B Olopade OI. A perfect storm: how tumor biology, genomics, and health care delivery patterns collide to create a racial survival disparity in breast cancer and proposed interventions for change. CA Cancer J Clin. (2015) 65:221–38. 10.3322/caac.21271 25960198

[49] Hunt BR Hurlbert MS. Black:white disparities in breast cancer mortality in the 50 largest cities in the United States, 2005-2014. Cancer Epidemiol. (2016) 45:169–73. 10.1016/j.canep.2016.07.018 27720130

[50] Rust G Zhang S Malhotra K Reese L McRoy L Baltrus P. Paths to health equity: local area variation in progress toward eliminating breast cancer mortality disparities, 1990-2009. Cancer (2015) 121:2765–2774. 10.1002/cncr.29405 25906833 PMC4540479

[51] Van Der Wees PJ Zaslavsky AM Ayanian JZ. Improvements in health status after Massachusetts health care reform. Milbank Q. (2013) 91:663–89. 10.1111/1468-0009.12029 24320165 PMC3876186

[52] Ali AM Schmidt MK Bolla MK Wang Q Gago-Dominguez M Castelao JE. Alcohol consumption and survival after a breast cancer diagnosis: a literature-based meta-analysis and collaborative analysis of data for 29,239 cases. Cancer Epidemiol Biomarkers Prev. (2014) 23:934–45. 10.1158/1055-9965.EPI-13-0901 24636975 PMC4542077

[53] Nechuta S Chen WY Cai H Poole EM Kwan ML Flatt SW. A pooled analysis of post-diagnosis lifestyle factors in association with late estrogen-receptor-positive breast cancer prognosis. Int J Cancer. (2016) 138:2088–2097. 10.1002/ijc.29940 26606746 PMC4764465

[54] Williams LA Olshan AF Tse CK Bell ME Troester MA. Alcohol intake and invasive breast cancer risk by molecular subtype and race in the Carolina Breast Cancer Study. Cancer Causes Control (2016) 27:259–69. 10.1007/s10552-015-0703-4 26705260 PMC5074055

[55] Swanson CA Coates RJ Malone KE Gammon MD Schoenberg JB Brogan DJ. Alcohol consumption and breast cancer risk among women under age 45 years. Epidemiology (1997) 8:231–7. 10.1097/00001648-199705000-00001 9115015

[56] Horn-Ross PL Canchola AJ West DW Stewart SL Bernstein L Deapen D. Patterns of alcohol consumption and breast cancer risk in the California Teachers Study cohort. Cancer Epidemiol Biomarkers Prev. (2004) 13:405–411. 15006916

[57] Hamajima N Hirose K Tajima K Rohan T Calle EE Heath CW Jr. Alcohol, tobacco and breast cancer–collaborative reanalysis of individual data from 53 epidemiological studies, including 58,515 women with breast cancer and 95,067 women without the disease. Br J Cancer (2002) 87:1234–45. 10.1038/sj.bjc.6600596 12439712 PMC2562507

[58] Jackson CL Hu FB Kawachi I Williams DR Mukamal KJ Rimm EB. Black-White differences in the relationship between alcohol drinking patterns and mortality among US men and women. Am J Public Health (2015) 105 (Suppl. 3):S534–43. 10.2105/AJPH.2015.302615 25905819 PMC4455501

[59] Kinney AY Millikan RC Lin YH Moorman PG Newman B. Alcohol consumption and breast cancer among black and white women in North Carolina (United States). Cancer Causes Control. (2000) 11:345–57. 10.1023/A:1008973709917 10843445

[60] Llanos AA Makambi KH Tucker CA Shields PG Adams-Campbell LL. Alcohol, anthropometrics, and breast cancer risk in African American women. Breast J. (2012) 18:394–5. 10.1111/j.1524-4741.2012.01265.x 22681594 PMC3760188

[61] Caetano R Herd D. Black drinking practices in northern California. Am J Drug Alcohol Abuse (1984) 10:571–87. 10.3109/00952998409001494 6534185

[62] Trichopoulos D Adami HO Ekbom A Hsieh CC Lagiou P. Early life events and conditions and breast cancer risk: from epidemiology to etiology. Int J Cancer (2008) 122:481–5. 10.1002/ijc.23303 18022897

[63] van't Veer P Kok FJ Hermus RJ Sturmans F. Alcohol dose, frequency and age at first exposure in relation to the risk of breast cancer. Int J Epidemiol. (1989) 18:511–7. 10.1093/ije/18.3.511 2807651

[64] Harvey EB Schairer C Brinton LA Hoover RN Fraumeni JF Jr. Alcohol consumption and breast cancer. J Natl Cancer Inst. (1987) 78:657–61. 3104648

[65] Young TB. A case-control study of breast cancer and alcohol consumption habits. Cancer. (1989) 64:552–8. 10.1002/1097-0142(19890715)64:2<552::AID-CNCR2820640233>3.0.CO;2-Y 2736501

[66] White AJ DeRoo LA Weinberg CR Sandler DP. Lifetime alcohol intake, binge drinking behaviors, and breast cancer risk. Am J Epidemiol. (2017) 186:541–9. 10.1093/aje/kwx118 28486582 PMC5860148

[67] Irwin ML. Weight loss interventions and breast cancer survival: the time is now. J Clin Oncol. (2014) 32:2197–99. 10.1200/JCO.2014.56.4583 24934785

[68] Dossus L Boutron-Ruault MC Kaaks R Gram IT Vilier A Fervers B. Active and passive cigarette smoking and breast cancer risk: results from the EPIC cohort. Int J Cancer (2014) 134:1871–88. 10.1002/ijc.28508 24590452

[69] Macacu A Autier P Boniol M Boyle P. Active and passive smoking and risk of breast cancer: a meta-analysis. Breast Cancer Res Treat (2015) 154:213–24. 10.1007/s10549-015-3628-4 26546245

